# Improving Temporal Coverage of an Energy-Efficient Data Extraction Algorithm for Environmental Monitoring Using Wireless Sensor Networks

**DOI:** 10.3390/s90604941

**Published:** 2009-06-23

**Authors:** Supriyo Chatterjea, Paul Havinga

**Affiliations:** Pervasive Systems Group, Faculty of Electrical Engineering, Mathematics and Computer Science, University of Twente, P.O. Box 217, 7500AE, Enschede, The Netherlands; E-Mail: havinga@cs.utwente.nl

**Keywords:** wireless sensor networks, environmental monitoring, adaptive sampling

## Abstract

Collecting raw data from a wireless sensor network for environmental monitoring applications can be a difficult task due to the high energy consumption involved. This is especially difficult when the application requires specialized sensors that have very high energy consumption, e.g. hydrological sensors for monitoring marine environments. This paper introduces a technique for reducing energy consumption by minimizing sensor sampling operations. In addition, we illustrate how a randomized algorithm can be used to improve temporal coverage such that the time between the occurrence of an event and its detection can be minimized. We evaluate our approach using real data collected from a sensor network deployment on the Great Barrier Reef.

## 1. Introduction

Wireless sensor networks (WSNs) are increasingly being used in a wide range of environmental monitoring applications. The primary reason for this is that WSNs are capable of monitoring various parameters at high spatial and temporal resolutions. Such fine-grained measurements cannot be taken using conventional data loggers.

However, collecting raw sensor readings from a large scale WSN can be a highly challenging task. The sensor nodes that make up a WSN are typically battery powered and communicate among themselves using a radio transceiver. A single sensor node may also have a host of sensors attached to it. As both the radio transceiver and the attached sensors consume significant amounts of energy, it is essential to use energy-efficient algorithms to ensure that the lifespan of the network is maximized. Transmitting large amounts of data can also lead to congestion and packet loss within the network, as sensor nodes usually have a very small bandwidth due to the low duty cycle of their transceivers. Thus in addition to energy-efficiency, any developed algorithm for data collection for WSNs should also maintain an acceptable level of data quality that meets the user's requirements.

In this paper, we first present an algorithm that minimizes energy consumption by not only reducing the number of sensor sampling operations but also by reducing message transmissions. The basic idea is to use time-series forecasting to try and predict future sensor readings. When the trend of a particular sensor reading is fairly constant and thus predictable, the sensor sampling frequency and message transmission rate are reduced. Conversely, when the trend changes, both the sampling rate and message transmission rates are increased. Additionally the paper describes how a randomized wake-up scheme can be used to improve temporal coverage so as to minimize the time that elapses between the occurrence of an event and its detection. The randomized algorithm eliminates the need for additional communication between nodes thus improving overall energy consumption. We show that using our randomized algorithm, in certain cases, the delay between the occurrence of an event and its detection can be reduced to just one epoch (Note: An epoch refers to the time period between two consecutive samples.)

The algorithm is evaluated using real-life sensor data that has been collected from a sensor network that has been deployed in the Great Barrier Reef, Australia. We first provide the reader with an overview of the sensor network deployment in the Great Barrier Reef. We then briefly outline the important concepts of time series forecasting that are relevant to our adaptive sampling algorithm. Next we provide the details of the basic adaptive sampling algorithm and then describe how the temporal coverage of the adaptive sampling algorithm can be improved. We then give an overview of the related work in this area and finally conclude the paper.

## 2. Application Scenario: Sensor Networking the Great Barrier Reef

The Great Barrier Reef (GBR), located along the north-east coast of Australia is made up of over 3,200 reefs and extends over an area of 280,000 km^2^. It is the world's largest coral reef system. The GBR however, is under threat due to the following factors:

- *Global warming*: A temperature rise of between 2 and 3 degrees celsius would result in 97% of the Great Barrier Reef being bleached every year [1].
- *Pollution*: Rivers flowing into the GBR in the north-east coast of Australia flow through large areas of farmland. Thus excess fertilisers and pesticides flow from these farmlands into the GBR [2].
- *Overfishing*: It is quite common for fishermen to catch unwanted species of fish. This can cause major disruptions to the food chain and thus eventually harm the corals in the GBR.

**Figure 1.**
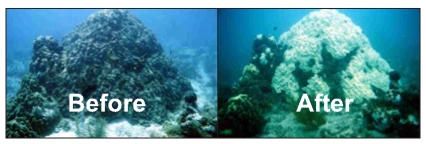
The effects of coral bleaching (Adapted from [3]).

These threats affect the GBR adversely in several ways. For example, overfishing reduces coral reef diversity and recovery. Surface runoff from agricultural lands causes algae blooms and Crown of thorn starfish outbreaks, which are known to destroy coral reefs. Thermal stress, which could be caused by global warming, causes *coral bleaching*. Coral bleaching is a stress condition that causes the breakdown of the symbiotic relationship between the corals and unicellular algae known as *zooxanthellae*. It is these microscopic plants that provide a coral with its normal healthy colour. When coral bleaching occurs, the algae are expelled from the coral tissue resulting in the corals becoming white. This is illustrated in Figure 1. While the corals do not initially die and can recover from coral bleaching, prolonged periods of stress can result in the eventual death of a coral.

In order to ensure the long-time survivability of the GBR, it is essential to understand the precise impact that global warming, pollution and over-fishing play in the destruction of the GBR. While global warming is a cause that cannot be readily controlled, pollution and overfishing are. Being able to monitor environmental parameters such as temperature, light, salinity, level of pollutants, etc. at real-time and at a high spatial and temporal resolution would enable scientists to better understand the underlying complex environmental processes that help shape the behaviour of the biological and physical characteristics of the GBR. As an example, if high levels of pollutants (e.g. pesticides) are detected in the GBR, farmers along the coast can be advised to reduce the amount of pesticides that are used.

We are currently working together with the Australian Institute of Marine Science (AIMS) [4] to set up a large-scale wireless sensor network to monitor various environmental parameters on the Great Barrier Reef (GBR) in Australia. Scientists at AIMS intend to use the collected data to study coral bleaching, reef-wide temperature fluctuations, impact of temperature on aquatic life and pollution.

One of the reefs under study is the Davies Reef which is approximately 80 km north-east of the city of Townsville in North Queensland, Australia. Currently, AIMS has a couple of data loggers situated on the reef that records temperature at two separate depths once every thirty minutes. Scientists from AIMS need to visit the reef periodically to download the data from the loggers.

The drawback of the current system is that it only allows single-point measurements. Thus it is impossible to get a true representation of the temperature gradients spanning the entire reef which is around 7 km in length. This is because the scale of the fluctuation of environmental parameters in the GBR, ranges from kilometre-wide oceanic mixing to millimetre-scale inter-skeletal currents. Also, the practice of collecting the data once every few weeks makes it impossible to study the trends of various parameters in real-time. Deploying a sensor network would not only allow high resolution monitoring in both the spatial and temporal dimensions but would also enable scientists to improve their understanding of the complex environmental processes by studying data streaming in from the reef in real-time.

**Figure 2.**
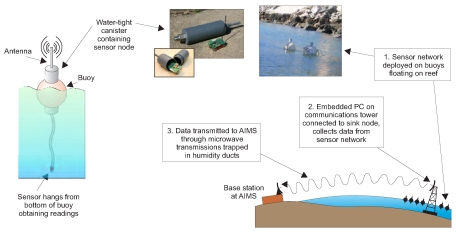
Overview of data collection system at Davies Reef.

**Figure 3.**
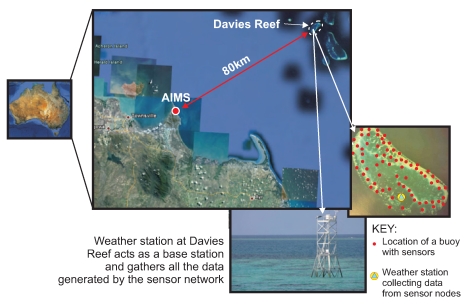
Data collected by the sensor network is transmitted to AIMS via a microwave link (Adapted from [7]).

The new data collection system that we are deploying at Davies reef can be broken down into three main components as shown in Figure 2:

- *Ambient μNodes*: These are the sensor nodes from Ambient Systems [5] that will be placed in water and shock-proof canisters and then placed in buoys around the reef.
- *Embedded PC*: An embedded PC will be placed on a communication tower and will act as the sink node collecting data from all the sensors in the reef.
- *Microwave link*: This will allow data to be transmitted from the Embedded PC to the AIMS base station 80 km away using microwave transmissions trapped inside humidity ducts that form directly above the surface of the sea [6]. This is illustrated in Figure 3.

The algorithm presented in this paper is designed for the first component, i.e.Ambient *μ*Nodes.

## 3. Preliminaries of Time-Series Forecasting

Time-series forecasting is a technique that has been used in a wide variety of disciplines such as engineering, economics, and the natural and social sciences to predict the outcome of a particular parameter based on a set of historical values. These historical values, often referred to as a “time series”, are spaced equally over time and can represent anything from monthly sales data to temperature readings acquired periodically by sensor nodes. Wei [8] and Brockwell and Davis [9] provide a very good introduction to time series forecasting.

The general approach to time-series forecasting can be described in four main steps:

1. Analyze the data and identify the existence of a trend or a seasonal component.
2. Remove the trend and seasonal components to get *stationary* (defined below) residuals. This may be carried out by applying a transformation to the data.
3. Choose a suitable model to fit the residuals.
4. Predict the outcome by forecasting the residuals and then inverting the transformations described above to arrive at forecasts of the original series.

Before describing details of how we perform each of the above steps in our data aggregation framework, we first present some basic definitions.

### Definition 3.1

Let *X_t_* be a time series where *t* = 1, 2, 3,… We define the mean of *X_t_* as,

(1) $${µ}_{t}=E\left(X_{t}\right)$$

### Definition 3.2

*Covariance* is a measure of to what extent two variables vary together. Thus the covariance function between *X_t_*_1_ and *X_t_*_2_ is defined as,

(2) $$\gamma \left(t_{1},t_{2}\right)=\text{Cov}\left(X_{t_{1}},X_{t_{2}}\right)=E\left[\left(X_{t_{1}}-\mu_{t_{1}}\right)\left(X_{t_{2}}-\mu_{t_{2}}\right)\right]$$

### Definition 3.3

We define the sample autocovariance at lag *h* of *X_t_* for *h* = 0,1,2,…,*T* as

(3) $$\gamma \left(h\right)=\frac{\sum_{t=h+1}^{T}\left(X_{t}-\bar{X}\right)\left(X_{t-h}-\bar{X}\right)}{T}$$

$\bar{X}=T^{-1}\sum_{t=1}^{T}X_{t}$ *X_t_* is the sample mean of the time series *X_t_*. Note that γ(0) is simply the variance of *X_t_*.

### Definition 3.4

The *autocorrelation function* (ACF), *ρ_h_*, which indicates the correlation between *X_t_* and *X_t_*_+_*_h_*, is

(4) $$\rho \left(h\right)=\frac{\gamma \left(h\right)}{\gamma \left(0\right)}$$

### Definition 3.5

We consider the time series *X_t_* to be *stationary* if the following two conditions are met:

(5) $$E\left(X_{t}\right)=\mu_{t}=\mu_{t+\tau }\forall \tau \in \mathbb{R}$$

(6) γ(t+h,t)=γ(t+h+τ,t+τ)∀τ∈ℝ

Equation 5 and Equation 6 imply that the mean and covariance remain constant over time respectively. In the case of Equation 6, the covariance remains constant for a given lag *h*.

### Definition 3.6

A process is called a *white noise* process if it is a sequence of uncorrelated random variables with zero mean and variance, *σ*^2^. We refer to white noise using the notation WN(0, *σ*^2^). By definition, it immediately follows that a white noise process is stationary with the autocovariance function,

(7) $$\gamma \left(t+h,t\right)=\{\begin{array}{cc} \sigma^{2} & \text{ifh}=0,\\ 0 & \text{ifh}\neq 0 \end{array}$$

### 3.1. Analysis of Data and Identification of Trend

As mentioned earlier, the first step is to identify the trend and seasonal component. However, as we make predictions using a small number of sensor readings taken over a relatively short period of time (e.g. 20 mins), we make the assumption that the readings do not contain any seasonal component. Instead, given that *t* represents time, we model the sensor readings, *R_t_* using a slowly changing function known as the *trend component, m_t_* and an additional stochastic component, *X_t_* that has zero mean. Thus we use the following model: *R_t_* = *m_t_* + *X_t_*.

The main idea is to eliminate the trend component, *m_t_*, from *R_t_* so that the behavior of *X_t_* can be studied. There are various ways of estimating the trend for a given data set, e.g. using polynomial fitting, moving averages, differencing, double exponential smoothing, etc. Due to the highly limited computation and memory resources of sensor nodes, we make use of a first degree polynomial, i.e. *m_t_* = *a*_0_ + *a*_1_*t*.

The coefficients *a*_0_, and *a*_1_ can be computed by minimizing the sum of squares, $Q=\sum_{t=1}^{T}{\left(R_{t}-m_{t}\right)}^{2}$. In order to find the values of *a*_0_ and *a*_1_ that minimize *Q*, we need to solve the following equations:

(8) $$\frac{\partial Q}{\partial a_{0}}=-2\sum_{t=1}^{T}\left(R_{t}-a_{0}-a_{1}t\right)=0$$

(9) $$\frac{\partial Q}{\partial a_{1}}=-2\sum_{t=1}^{T}\left(R_{t}-a_{0}-a_{1}t\right)t=0$$

Solving Equations 8 and 9 leads to:

(10) $$a_{0}=\frac{\sum_{t=1}^{T}\left(t-\bar{t}\right)\left(R_{t}-\bar{R}\right)}{\sum_{t=1}^{T}{\left(t-\bar{t}\right)}^{2}}$$

(11) $$a_{1}=\bar{R}-a_{0}\bar{t}$$

**Figure 4.**
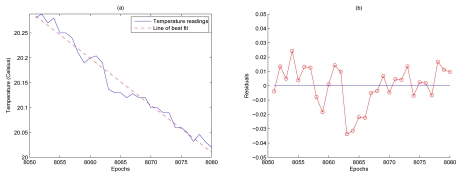
Temperature sensor readings and the corresponding residuals (Data obtained from Nelly Bay in the GBR [10]).

Eliminating the trend component from the sensor readings results in the residuals. This is shown in Figure 4(b). Note that the residuals have been obtained from the difference between the temperature sensor readings and the line of best fit illustrated in Figure 4(a). The residuals display two distinct characteristics. Firstly, there is no noticeable trend and secondly there are particular long stretches of residuals that have the same sign. This would be an unlikely occurrence if the residuals were observations of white noise with zero mean. This smoothness naturally indicates a certain level of dependence between readings [9]. The algorithm in this paper studies this dependence characteristic and uses it to help understand the behavior of the residuals so that predictions can be made.

Now that a stationary time series has been obtained, the next step is to choose an appropriate model that can adequately represent the behavior of the time series.

Stationary processes can be modelled using *autoregressive moving average* (ARMA) models. The ARMA model is a tool for understanding and subsequently predicting future values of a stationary series. The model consists of an autoregressive part, AR and a moving average part, MA. It is generally referred to as the ARMA(*p, q*) model where *p* is the order of autoregressive part and *q* is the order of the moving average part. The AR(*p*) model is essentially a linear regression of the current value of the series against *p* prior values of the series, *X_t_*_−1_, *X_t_*_−2_,…, *X_t_*_−_*_p_*. The MA model on the other hand is a linear regression of the current value of the series against the white noise of one or more prior values of the series, *Z_t_*_−1_, *Z_t_*_−2_,…, *Z_t_*_−_*_p_*. The complete ARMA(*p, q*) model is defined as follows,

(12) $$X_{t}=\phi_{1}X_{t-1}+\ldots +\phi_{p}X_{t-p}+Z_{t}+\theta_{1}Z_{t-1}+\ldots +\theta_{q}Z_{t-q}$$

*Z_t_* ∼WN(0, *σ*^2^) and *ϕ_i_, i* = 1,2, …, *p* and *θ_i_, i* = 1, 2,…, *q* are constants [9].

However, due to the limited computation and memory resources on a sensor node, we use an AR(1) model instead of the full ARMA model (i.e. *q* = 0) to predict the value *R_t_*, i.e. *X_t_* = *ϕ*_1_*X_t_*_−1_ + *Z_t_*. The constant *ϕ*_1_ can now be estimated using the Yule-Walker estimator, i.e. ${\hat{\phi }}_{1}={\hat{\rho }}_{1}=\frac{\gamma \left(1\right)}{\gamma \left(0\right)}$ [8]. We can then state that the general form of the minimum mean square error *m*-step forecast equation is

(13) $${\hat{X}}_{t+m}=\mu +\phi^{m}\left(X_{t}-\mu \right),m\geq 1$$

## 4. Prediction Using the Adaptive Sampling Algorithm

In this section, we describe the original adaptive sampling algorithm which was first introduced in [11]. The algorithm uses the time-series forecasting concepts described in the previous section to predict sensor readings in the future.

In general terms, when the reading of a particular sensor on a node can be predicted based on the recent past, we reduce the frequency of sampling the sensors by skipping a number of sensor sampling operations and performing predictions instead. However, the moment the prediction differs from the actual sampled reading by an amount specified by the user, the sampling frequency is increased. This local prediction mechanism also helps reduce the number of sensor readings that need to be transmitted to the sink node. Algorithm 1 gives a detailed description of how adaptive sampling is performed. Table 1 lists the definition of the acronyms mentioned in Algorithm 1. Note that sensor power-up times are not considered in this algorithm as a user cannot set a minimum epoch that is lesser than the sensor power-up time.

**Algorithm 1** Adaptive sampling1:**repeat**2: *R_A,t_* ← Acquire sensor reading at current time, t3: Append *R_A,t_* to BUFFER4: *t* = *t* +15:**until** size(BUFFER)=FULL6:**repeat**7: **if***SS* = 0 **then**8:  *R_A,t_* ← Acquire sensor reading at current time, t9:  *R_F,t_* ← Forecast reading for current time, t based on contents of BUFFER10:  **if** |*R_A,t_ − R_F,t_*| < *δ***then**11:   **if***CSSL* < *MSSL***then**12:    *CSSL* ← *CSSL*+113:   **end if**14:   **if***SS* < *CSSL***then**15:    *SS* < *SS* +116:   **end if**17:  **else**18:   *SS* ← 019:  **end if**20:  Remove oldest reading from BUFFER21:  Append *R_A,t_* to BUFFER22:  Use *R_A,t_* and previously sampled reading to linearly interpolate intermediate samples if any23: **else**24:  *SS* < *SS* − 125:  *R_F,t_* ← Forecast reading for current time, t based on contents of BUFFER26:  Remove oldest reading from BUFFER27:  Append *R_F,t_* to BUFFER28: **end if**29: *t* = *t* + 130:**until** StopDataCollection = TRUE

## 5. Improving Temporal Coverage

Our earlier work (which we refer to as *adaptive sampling* or *AS* from this point onward) focussed on energy consumption and data quality in terms of accuracy of the collected sensor readings, it did not discuss the issue of *temporal coverage*. By coverage we refer to the maximum delay between the occurrence and detection of any event.

If sensor readings follow a predictable trend for long periods of time, the drawback of the *AS* algorithm is that the value of *SS* tends to remain at *MSSL*. While this is highly beneficial in terms of reducing energy consumption, an obvious drawback is that there is a greater chance of missing an event that might begin during the period when sensor sampling operations are skipped, i.e. *SS* > 0.

In order to reduce the time taken between the occurrence of an event and its detection, we illustrate how we take advantage of the spatial correlation that exists between neighbouring sensors and introduce a randomized scheme that staggers the sampling times of adjacent nodes. This effectively decreases the chances of *SS* reaching *MSSL*.

In order to justify our approach we first illustrate how sensor readings between adjacent nodes can be correlated. We then proceed to show how the readings of neighbouring sensors can be used to improve temporal coverage. This approach is then further improved by using a randomized algorithm that further improves temporal coverage.

### 5.1. Correlation of Sensor Readings of Adjacent Sensor Nodes

**Figure 5.**
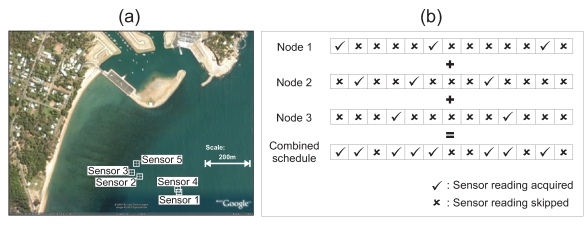
(a) Sensors deployed in Nelly Bay, Great Barrier Reef, Australia; (b) Combining schedules of adjacent nodes helps improve temporal coverage.

**Figure 6.**
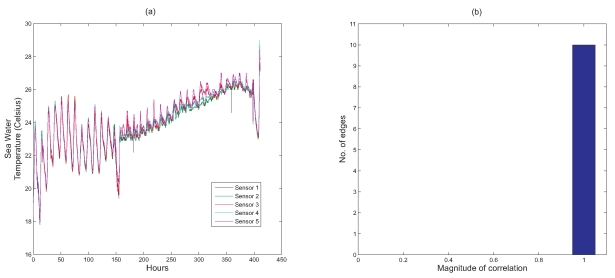
(a) Temperature readings from sensors deployed in Nelly Bay, Great Barrier Reef, Australia over a period of 17 days; (b) Number of edges with correlation close to 1.

Figure 5 illustrates a deployment of five sensors in Nelly Bay in the Great Barrier Reef. As can be seen from the figure, the maximum distance between the sensors is around 350 m. We make the assumption that all sensors are within radio communication range of each other. This implies that the five sensors form a fully connected graph with a total of 10 edges. Note that each edge represents the correlation that exists between the two nodes at either end of the edge. Figure 6(a) shows the temperature readings collected from all the five sensors over a period of around 17 days.

Next, we use Figure 6(b) to illustrate how every node's readings are correlated with every one of its adjacent neighbours. The histogram in Figure 6(b) shows that all 10 edges have correlation values that are close to 1. From this, we can conclude that if a particular node detects a certain event, there will be a high probability that the adjacent nodes will also detect this event.

**Figure 7.**
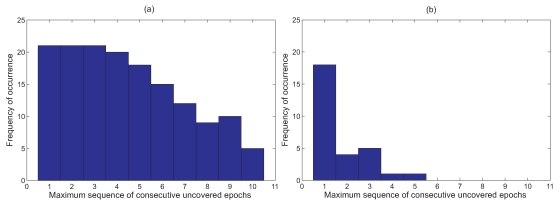
(a) Histogram showing spread of maximum sequence of consecutive uncovered epochs for *AS* algorithm; (b) Histogram showing maximum sequence of consecutive uncovered epochs when spatial correlations of adjacent nodes is considered.

As the *AS* algorithm disregards this spatial correlation between adjacent nodes, the temporal coverage of a particular node is only attributed to the node's own sampling frequency. In other words, if a node skips *x* samples, it is assumed that any event that occurs during these *x* epochs will not be detected. Figure 7(a) shows a histogram of the frequency of the maximum sequence of consecutive uncovered epochs that are attained by all the five nodes in the network based on the data set from Nelly Bay collected over 17 days.

However, if the sampling schedules of all adjacent nodes are combined, then the probability of all sensor nodes within a single hop missing an event due to a large *SS* value is greatly diminished. Figure 5 illustrates this concept. We can see clearly from Figure 7(b) that not only are long sequences of uncovered epochs completely eliminated, but the area of the graph in Figure 7(b) is significantly lower than that of Figure 7(a). This implies that combining sampling schedules greatly reduces the chances of missing an event.

In order to improve temporal coverage even further, we introduce a randomized scheme in addition to combining sampling schedules as mentioned above. The main motivation is that in certain instances, especially when the trend of sensor readings is changing very gradually and there is a strong spatial correlation between the readings of adjacent nodes, nodes close to each other tend to have similar *SS* values. This essentially means that the sampling times of sensors which are close to one another may be quite synchronized. This is undesirable as even if the sampling schedules are combined, their resulting schedule remains relatively unchanged as compared to the schedules of individual nodes.

**Figure 8.**
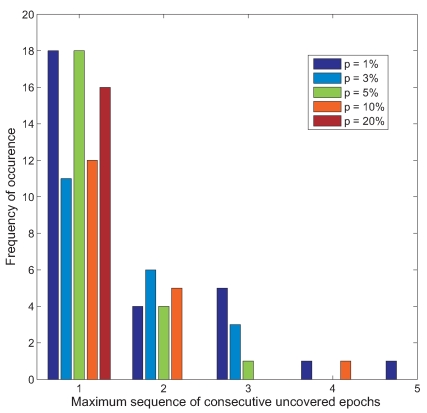
Histogram showing spread of maximum sequence of consecutive uncovered epochs when combining schedules of adjacent nodes using randomized scheme for different values of *p*.

To prevent this from occurring, we introduce a scheme where every time a node has a *SS* value greater than zero, i.e. it is supposed to skip the sensor sampling operation, the *SS* value is reset to zero with probability *p*. This reduces the chance of having synchronized sampling schedules between adjacent sensor nodes. In our simulations, we vary the value of *p* from 1% to 20%. This is shown in Figure 8. It can be seen that as *p* is increased, the maximum sequence of uncovered epochs is greatly reduced. In fact when *p* is set to 20%, the delay between the occurrence of an event and its detection is only one epoch. Thus, in such cases, if the duration of an event is several epochs long, none of the events will be missed. This is quite a likely scenario in the GBR setting since very often, an event which occurs generally lasts a while.

## 6. Related Work

A wide variety of techniques can be found that deal with extracting data in an energy-efficient manner. The authors in [12] describe a technique to prevent the need to sample sensors in response to an incoming query. However, the technique is not able to cope with sudden changes in the correlation models and also fails to recognize the importance of temporal fluctuations in these models. KEN [13] is able to create and adapt models on the fly and thus adapt but does not describe how to deal with topology changes. Both PAQ [14] and SAF [15] are similar to our scheme in the sense that they also use time-series forecasting to identify temporal correlations within the network itself. There are a number of papers [16, 17] which also model the sensed data using some sort of a linear model. Just as we take advantage of the spatial correlations between adjacent sensor nodes, the authors in [18] also identify nodes which have similar readings and then choose certain representative nodes to which are chosen to transmit the sensed data.

However, all the schemes mentioned above only deal with reducing message transmissions. While this is helpful, this is far from adequate when the application uses sensors which consume a lot of energy. Note that many sophisticated sensors used for environmental monitoring also have long start-up and sampling times. This too has a large impact on energy consumption. To our knowledge, we are not aware of any other work which deals with reducing the duty cycle of the sensors themselves.

Another major difference between our work and the schemes mentioned above, is that our scheme works on only *partially* available data since we do not sample every sensor reading but skip a large number of samples to save energy. All the above schemes build models or make decisions using sensor readings obtained at every epoch.

## 7. Conclusions and Future Work

We have presented an adaptive sensor sampling scheme that takes advantage of temporal correlations of sensor readings in order to reduce energy consumption. While the scheme can be used in various environmental monitoring scenarios, we have focussed on a deployment of sensor nodes on the Great Barrier Reef in Australia as the deployment involves using sensors that consume a lot of energy. Such a deployment would benefit greatly from using an adaptive sampling scheme. The original problem with the adaptive sampling scheme was that there was a very high probability of missing certain events as sensors were switched off for extended durations. This paper illustrates how temporal coverage can be improved by combining schedules of nodes next to each other. We also demonstrate how results can be further improved by using a randomized scheme to combine schedules without incurring additional overheads due to communication. The randomized scheme shows how the time between event occurrence and event detection may be reduced to just one epoch in certain scenarios.

Our results in Figure 6(b) show the existence of a strong correlation between adjacent sensor nodes and our technique improves temporal coverage by taking advantage of this fact. However, it should be noted that this is not always the case, i.e. adjacent nodes may not always have correlated sensor readings. In such instances, the strategy of combining sampling schedules would not work.In order to address this issue, we are currently designing a distributed algorithm, where every node first decides whether its readings are correlated with a particular adjacent node based on the recent history of sampled readings. The algorithm to improve temporal coverage presented in this paper is *only* used if a correlation has been identified. Thus, this technique will allow the nodes to run at their optimal level regardless of the existence of any spatial correlation between sensor readings of adjacent nodes.

## Figures and Tables

**Table 1.** Description of acronyms used in Algorithm 1.

Acronym	Full form	Description

*SS*	Skip Samples	*SS* indicates the number of samples that should be skipped before the next sensor reading is acquired. *SS* is decremented by one every time a sample is skipped.
*CSSL*	Current Skip Samples Limit	Every time *SS* reaches 0, it starts decrementing from a starting value of *CSSL. CSSL* is incremented by one every time the difference between the forecast and an acquired reading is within the user-defined error threshold, *δ*. Otherwise it is reset to 0.
*MSSL*	Maximum Skip Samples Limit	*CSSL* can reach a maximum value of *MSSL*.

## References

[1] Jones R. (2003). Managing climate change risks. OECD Workshop on the Benefits of Climate Policy: Improving Information for Policy Makers.

[2] Death G., Lough J., Fabricius K. (2009). Declining coral calcification on the great barrier reef. Science.

[3] (2008). Coral bleaching - a sign of the times. http://www.marinephotobank.org/photocenter/stories_coralbleach.php.

[4] AIMS Reef at our fingertips. http://www.aims.gov.au/pages/about/communications/waypoint/headlines-04.html.

[5] Ambient Systems http://www.ambient-systems.net/ambient/index.htm.

[6] Palazzi C., Woods G., Atkinson I., Kininmonth S. (2005). High speed over ocean radio link to great barrier reef. TENCON. IEEE.

[7] Google earth (2008). http://earth.google.com/.

[8] Wei W.W.S. (1990). Time Series Analysis.

[9] Brockwell P.J., Davis R.A. (1991). Introduction to Time-Series and Forecasting.

[10] Bondarenko O., Kininmonth S., Kingsford M., Palaniswami M., Marusic S., Law Y.W. (2007). Underwater sensor networks, oceanography and plankton assemblages.

[11] Chatterjea S., Havinga P.J.M., Nikoletseas S.E., Chlebus B.S., Johnson D., Krishnamachari B. (2008). An adaptive and autonomous sensor sampling frequency control scheme for energy-efficient data acquisition in wireless sensor networks.

[12] Deshpande A., Guestrin C., Madden S., Hellerstein J.M., Hong W. (2005). Model-based approximate querying in sensor networks. VLDB J..

[13] Chu D., Deshpande A., Hellerstein J.M., Hong W. (2006). Approximate data collection in sensor networks using probabilistic models. ICDE.

[14] Tulone D., Madden S. (2006). Paq: Time series forecasting for approximate query answering in sensor networks. EWSN.

[15] Tulone D., Madden S. (2006). An energy-efficient querying framework in sensor networks for detecting node similarities.

[16] Emekci F., Tuna S.E., Agrawal D., El Abbadi A. (2005). Using linear models to monitor the physical world with sensors.

[17] Deligiannakis A., Kotidis Y., Roussopoulos N. (2004). Compressing historical information in sensor networks.

[18] Kotidis Y. (2005). Snapshot queries: Towards data-centric sensor networks.

